# Elucidation and reconstitution of hydrolyzable tannin biosynthesis

**DOI:** 10.5511/plantbiotechnology.24.0601a

**Published:** 2024-09-25

**Authors:** Ko Tahara, Carsten Milkowski, Chihiro Oda-Yamamizo

**Affiliations:** 1 Department of Forest Molecular Genetics and Biotechnology, Forestry and Forest Products Research Institute (FFPRI), 1 Matsunosato, Tsukuba, Ibaraki 305-8687, Japan; 2 Martin Luther University Halle-Wittenberg, AGRIPOLY: International Graduate School in Agricultural and Polymer Sciences, Betty-Heimann-Straße 3, D-06120 Halle, Germany

**Keywords:** biosynthetic pathway, ellagitannin, gallic acid, gallotannin, metabolic engineering

## Abstract

Hydrolyzable tannins (HTs) are a class of polyphenols produced mostly in core eudicot plants. They accumulate in various plant tissues and are considered to function as defense compounds that protect against herbivory, infections, and toxic metals (specifically aluminum ions). Moreover, HTs have industrial and pharmaceutical uses that benefit humans. Elucidating and reconstituting the biosynthesis of HTs is necessary for genetically engineering in planta functions and for efficiently producing HTs for human use. The biosynthesis of HTs is initiated by the formation of gallic acid from the shikimate pathway intermediate 3-dehydroshikimic acid, which is catalyzed by bifunctional dehydroquinate dehydratase/shikimate dehydrogenases (DQD/SDHs). In the second step, UDP glycosyltransferases (UGTs) esterify gallic acid with glucose to form β-glucogallin (1-*O*-galloyl-β-D-glucose). β-glucogallin is then converted to 1,2,3,4,6-penta-*O*-galloyl-β-D-glucose through a series of galloylation steps that are catalyzed by galloyltransferases, using β-glucogallin as a galloyl donor. Laccases subsequently catalyze the oxidative coupling between adjacent galloyl groups to form hexahydroxydiphenoyl (HHDP) groups, which are characteristic components of ellagitannins. Furthermore, monomeric ellagitannins can undergo oligomerization via intermolecular oxidative coupling, which is also catalyzed by laccases. To reconstitute the HT biosynthetic pathway in HT-non-accumulating plants, *DQD*/*SDH*s and *UGT*s from *Eucalyptus camaldulensis* were heterologously co-expressed in *Nicotiana benthamiana* leaves, which resulted in the production of gallic acid and β-glucogallin. In future studies, this transgenic system will be used to identify genes encoding galloyltransferases and laccases to further elucidate and reconstitute the HT biosynthetic pathway.

## Introduction

Plant tannins are water-soluble polyphenolic compounds that can bind and/or precipitate proteins and metals (Hagerman et al. 1997; Zhang et al. 2023). The term “tannin” is derived from “tanning”, which refers to the process of converting animal hides to leather using oak bark infusions. Humans have been using tannins as tanning agents since ancient times (Falcão and Araújo 2018). In East Asia, tannins have long been used as the active components of traditional medicines and in dyes (Okuda and Ito 2011; Okuda et al. 2009; Yoshida et al. 2004). The utility of tannins is due to their ability to interact with proteins and metals as well as their antioxidant activities. Tannins can be divided into two distinct groups according to their chemical structure: hydrolyzable tannins (HTs) and proanthocyanidins (PAs; also known as condensed tannins) (Seigler 1995). More specifically, HTs comprise polyphenolic carboxylic acids, such as gallic acid and its oxidative metabolites, that are esterified with a polyalcohol core, which is usually glucose. These tannins can be hydrolyzed into polyphenolic acids and a polyalcohol via acid, alkali, or enzyme treatments. In contrast, PAs are polymers formed through the condensation of flavan-3-ols, where the adjacent subunits are connected via C-C bonds, and they are not susceptible to hydrolysis.

HTs accumulate in various plant tissues, including leaves, bark, and roots, with relatively high concentrations (up to 70% of the dry weight), especially in long-lived woody plants (e.g., Haslam 2007; Scalbert et al. 1988; Tahara et al. 2014). In terms of their physiological functions, plant HTs are believed to contribute to chemical defense against herbivorous insects and mammals (Agrawal et al. 2012; Barbehenn and Constabel 2011; Takahashi and Shimada 2008) as well as phytopathogenic microbes and viruses (Buzzini et al. 2008; Puljula et al. 2020). The functions of chemical defense are supported by the negative associations between HT levels in forage and herbivore performance. The mechanisms that explain these functions could include the HT-induced production of reactive oxygen species at high pH in insect guts and decreased protein digestion due to HT-protein interactions (Barbehenn and Constabel 2011). We have proposed a new function of HTs. According to our studies on *Eucalyptus camaldulensis*, HTs may help detoxify toxic metals, specifically aluminum ions (Al), in plants. *E. camaldulensis* is a tree species that is highly resistant to Al stress, which is a primary factor limiting plant growth in acidic soils (Tahara et al. 2008). We determined that a distinct HT (oenothein B) in *E. camaldulensis* can bind with and detoxify Al in the root symplast (Tahara et al. 2014). The multiple *ortho*-diphenolic groups of oenothein B likely serve as Al-binding sites, enabling the formation of soluble 1 : 1 and 3 : 2 Al–oenothein B complexes as well as other insoluble complexes with Al (Tahara et al. 2017; Zhang et al. 2016). HTs have various pharmaceutical and industrial uses. For example, they are included in antidiarrheal drugs and corrosion inhibitors. The HTs in food, including fruits (e.g., pomegranates and strawberries), are of interest because of their antioxidant and antitumor activities, which may have beneficial effects on human health (Ito 2011; Ito et al. 2014). To understand and regulate HT functions in plants through genetic engineering and to establish efficient HT production systems, the molecular mechanism underlying HT biosynthesis must be elucidated.

**Figure 1:**
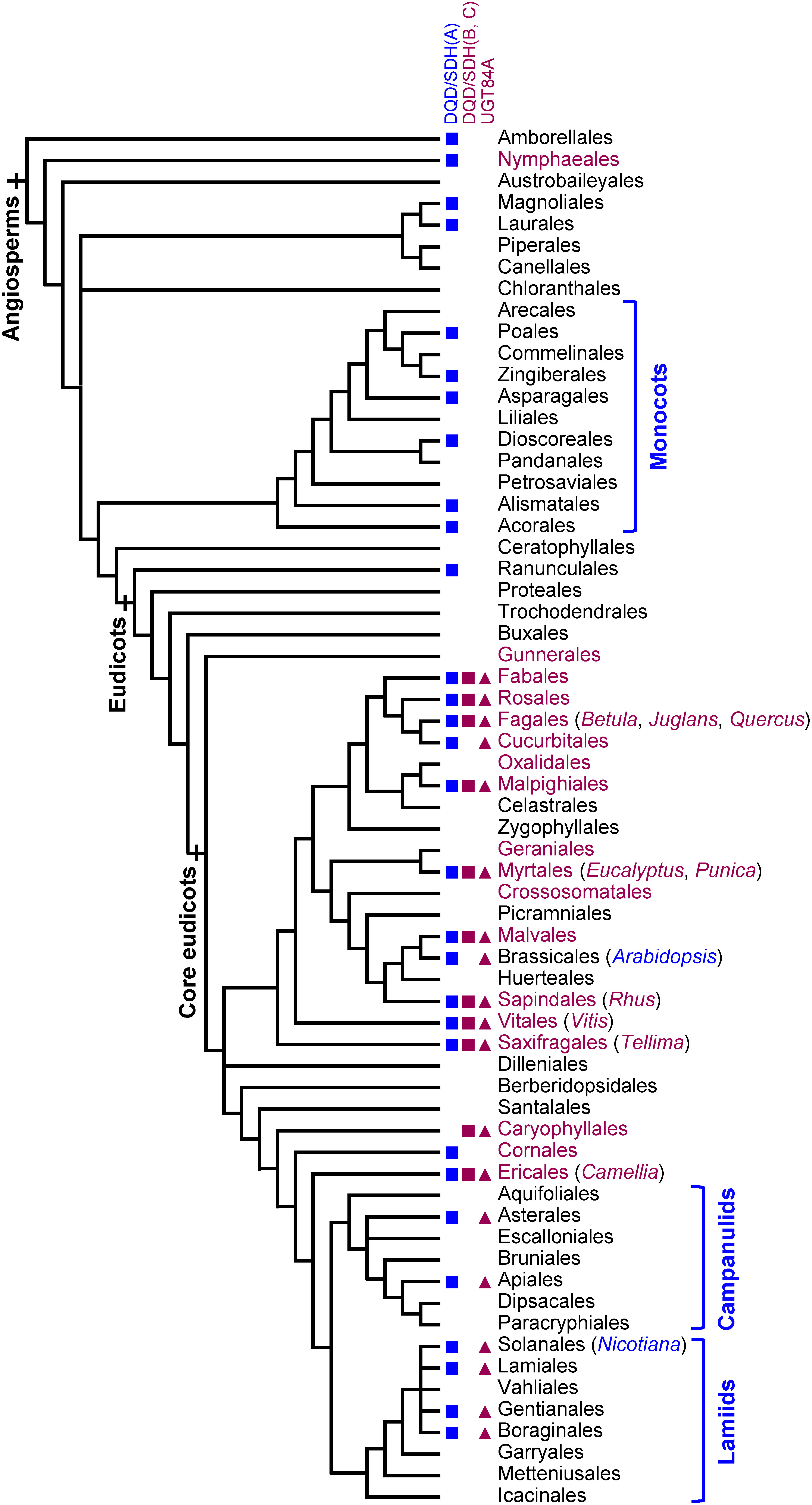
Figure 1. Distribution of hydrolyzable tannins and their biosynthetic enzymes in flowering plants. Orders (and genera) in which HTs were reportedly identified are indicated in magenta. The phylogenetic tree was constructed on the basis of the Angiosperm Phylogeny Group classification (APG IV). Blue squares indicate orders with DQD/SDH phylogenetic group A members, and magenta squares indicate orders with DQD/SDH phylogenetic group B or C members (Supplementary Figure S1). Magenta triangles indicate orders with UGTs that are grouped into the same clade as UGT84As (Supplementary Figure S2). This figure was adapted from Supplementary Figure S2 of a published article (Oda-Yamamizo et al. 2023).

Among tannins, PAs evolutionarily preceded HTs and are widely distributed in modern vascular plant species, from ferns to gymnosperms and angiosperms (Seigler 1995). Because of this and the long-standing scientific and economic interest in the related group of flavonoids, PA biosynthesis has been more thoroughly investigated and characterized than HT formation (Yu et al. 2023). Unlike PAs, HTs are restricted to angiosperms, and mainly to a specific group within core eudicots (Figure 1; Okuda et al. 2000). The herbaceous model plants often used for studies on metabolism, such as *Arabidopsis thaliana*, Solanaceae species, and monocot crops, are not included in the HT-accumulating taxonomic orders. This helps to explain why the genetic basis of the HT biosynthetic pathway remains largely unknown, although enzymatic studies by Gross and his group have deepened our understanding at the protein level (for review, see Gross 2009; Niemetz and Gross 2005). However, advances in sequencing and analytical chemistry technologies have facilitated the elucidation of the HT biosynthetic pathway at the genetic level using HT-accumulating non-model plants, including *Quercus robur* (e.g., Mittasch et al. 2014), *E. camaldulensis* (Oda-Yamamizo et al. 2023), and *Punica granatum* (Chang et al. 2021).

**Figure 2:**
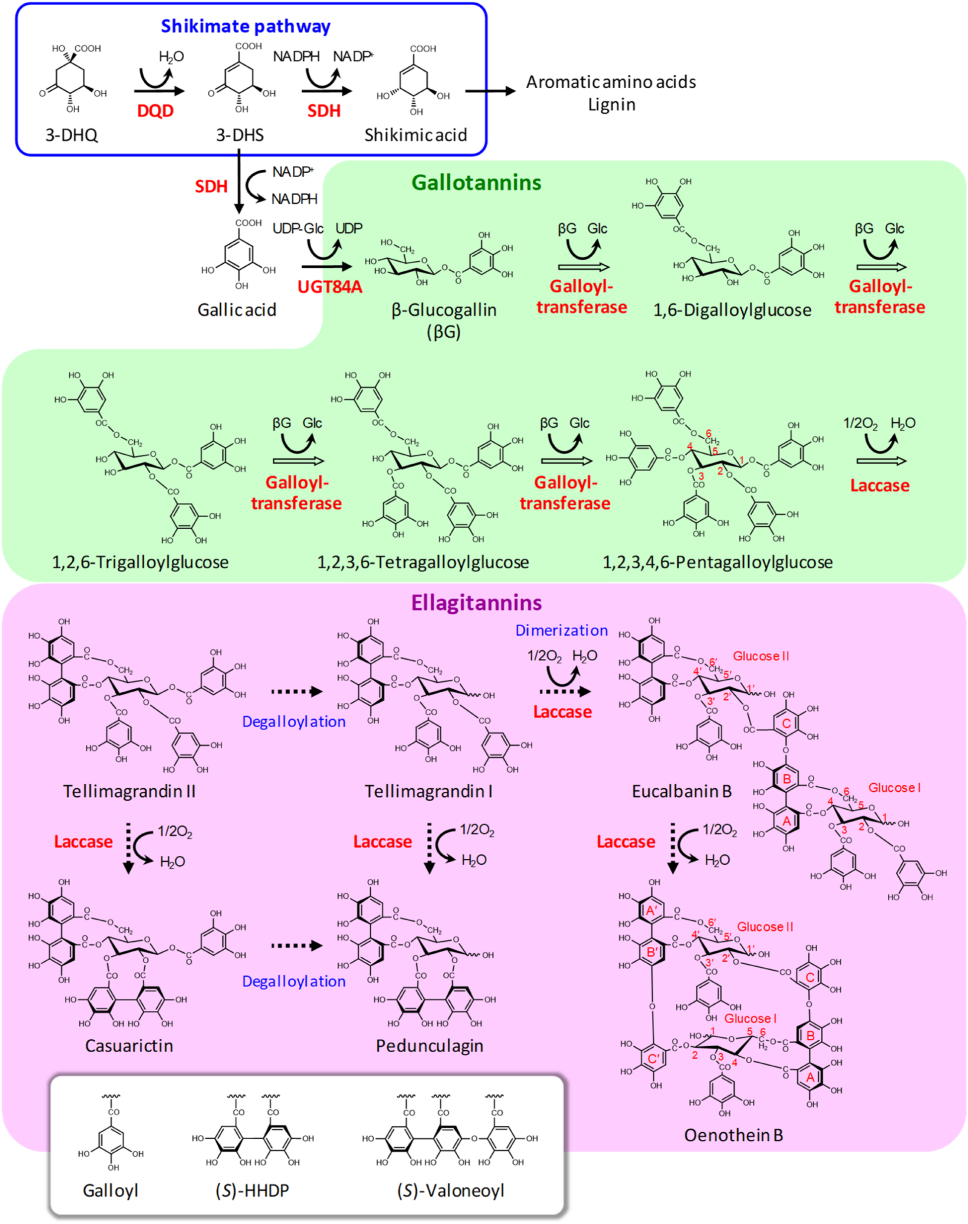
Figure 2. Putative hydrolyzable tannin biosynthetic pathway in *Eucalyptus camaldulensis*. Functionally characterized plant genes and enzymes are marked by a solid arrow. Hollow arrows indicate reactions catalyzed by partially purified enzymes with no available information regarding the corresponding genes. Dashed arrows indicate reactions catalyzed by unknown enzymes or genes that remain to be identified. 3-DHS, 3-dehydroshikimic acid; 3-DHQ, 3-dehydroquinic acid; DQD, dehydroquinate dehydratase; SDH, shikimate dehydrogenase; UGT, UDP-glycosyltransferase.

HTs have been divided into two subgroups: gallotannins and ellagitannins (Hagerman et al. 1997; Figure 2). Gallotannins, including galloylglucoses, release gallic acid upon hydrolysis, whereas ellagitannins contain a hexahydroxydiphenoyl (HHDP) group and produce ellagic acid when hydrolyzed. The HT biosynthetic pathway, which starts with the formation of gallic acid, branches off from the shikimate pathway (Niemetz and Gross 2005). Gallic acid is esterified with glucose to form β-glucogallin (1-*O*-galloyl-β-D-glucopyranose), which is converted to 1,2,3,4,6-pentagalloylglucose (1,2,3,4,6-penta-*O*-galloyl-β-D-glucopyranose) through a series of galloylation steps. Subsequently, ellagitannins are derived from 1,2,3,4,6-pentagalloylglucose by the oxidative coupling of adjacent galloyl moieties to yield HHDP groups. Galloyl and HHDP substituents can then be converted to other acyl groups, including digalloyl and dehydrohexahydroxydiphenoyl (DHHDP) groups (Okuda et al. 2009). Furthermore, monomeric ellagitannins are oligomerized by intermolecular oxidative coupling. The combination of various acyl group types and positions and several degrees of oligomerization results in a wide variety of HTs. To date, more than 500 HTs with diverse chemical structures have been identified (Yoshida et al. 2010). The types of HTs that accumulate preferentially in distinct plant species are related to their position in the evolutionary tree (Okuda et al. 2000; Yoshida et al. 2010). In this review on the HT biosynthetic pathway, we focus on the production of the dimeric ellagitannin oenothein B, which is one of the main HTs in *E. camaldulensis*, as well as on the recent advancements in the identification of the associated genes. We also outline our attempts to reconstitute the HT biosynthetic pathway in plants that do not naturally accumulate HTs.

## Biosynthesis of gallic acid

Retrobiosynthetic studies and analyses of the natural isotope abundance in *Rhus typhina* leaves revealed that gallic acid is formed by the dehydrogenation of the shikimate pathway intermediate 3-dehydroshikimic acid (3-DHS) (Werner et al. 1997, 2004; Figure 2). In the shikimate pathway, bifunctional dehydroquinate dehydratase/shikimate dehydrogenases (DQD/SDHs; EC 4.2.1.10/EC 1.1.1.25) catalyze the dehydration of 3-dehydroquinate (3-DHQ) to 3-DHS and the reduction of 3-DHS to shikimic acid (i.e., the “classical” SDH reaction) (Bischoff et al. 2001; Peek and Christendat 2015). Ossipov et al. (2003) partially purified a dehydroshikimate dehydrogenase (DSDG) that catalyzes the NADP^+^-dependent conversion of 3-DHS to gallic acid from birch (*Betula pubescens*) leaves. Genome analyses showed that many seed plants contain genes encoding DQD/SDH enzymes with diverse functions (Carrington et al. 2018; Guo et al. 2014). Muir et al. (2011) described a DQD/SDH from *Juglans regia* (JrSDH) that catalyzes the oxidation of 3-DHS to gallic acid. Bontpart et al. (2016) determined that VvSDH3 and VvSDH4 in *Vitis vinifera* convert 3-DHS to gallic acid, which is accompanied by a decrease in the “classical” SDH activity. The overexpression of *VvSDH3* in *V. vinifera* hairy roots results in increased levels of gallic acid, β-glucogallin, and galloylated flavan-3-ols, indicating VvSDH3 mediates gallic acid formation in planta. Huang et al. (2019) described CsDQD/SDHa and CsDQD/SDHd as two candidate enzymes that may contribute to gallic acid biosynthesis in *Camellia sinensis* (Jiang et al. 2013). Of the four DQD/SDH enzymes that were recently identified in *E. camaldulensis* (Tahara et al. 2021), EcDQD/SDH2 and 3 catalyze gallic acid formation in vitro, but also have residual DQD and “classical” SDH activities. Similar to VvSDH3 and 4 and DSDG from birch, EcDQD/SDH2 and 3 exhibit a strong preference for NADP^+^ over NAD^+^ as a cofactor for gallic acid formation. When NADP^+^ was replaced by NAD^+^, EcDQD/SDH2 did not produce gallic acid from 3-DHS, whereas EcDQD/SDH3 produced only trace amounts (Tahara et al. 2021). This cofactor preference is consistent with the sequence motif N-R-T at position 483-485 in EcDQD/SDH2 and 3 (Gritsunov et al. 2018; Peek and Christendat 2015; Tahara et al. 2021). As another remarkable feature shared with characterized gallic acid-forming DQD/SDH enzymes, EcDQD/SDH2 and 3 require high pH values above pH 9.0 for efficient gallic acid formation in vitro. In silico predictions localized EcDQD/SDH2 and 3 to plastids. Structural models suggest that EcDQD/SDH2 and 3 catalyze gallic acid formation via the reorientation of the substrate 3-DHS in the catalytic center (Singh and Christendat 2006, 2007; Tahara et al. 2021). In *E. camaldulensis*, *EcDQD*/*SDH2* and *3* are co-expressed with *UGT84A25* and *UGT84A26*, which encode UDP-glycosyltransferases involved in the formation of β-glucogallin (Tahara et al. 2018). This suggests that EcDQD/SDH2 and 3 can produce gallic acid in planta.

**Figure 3:**
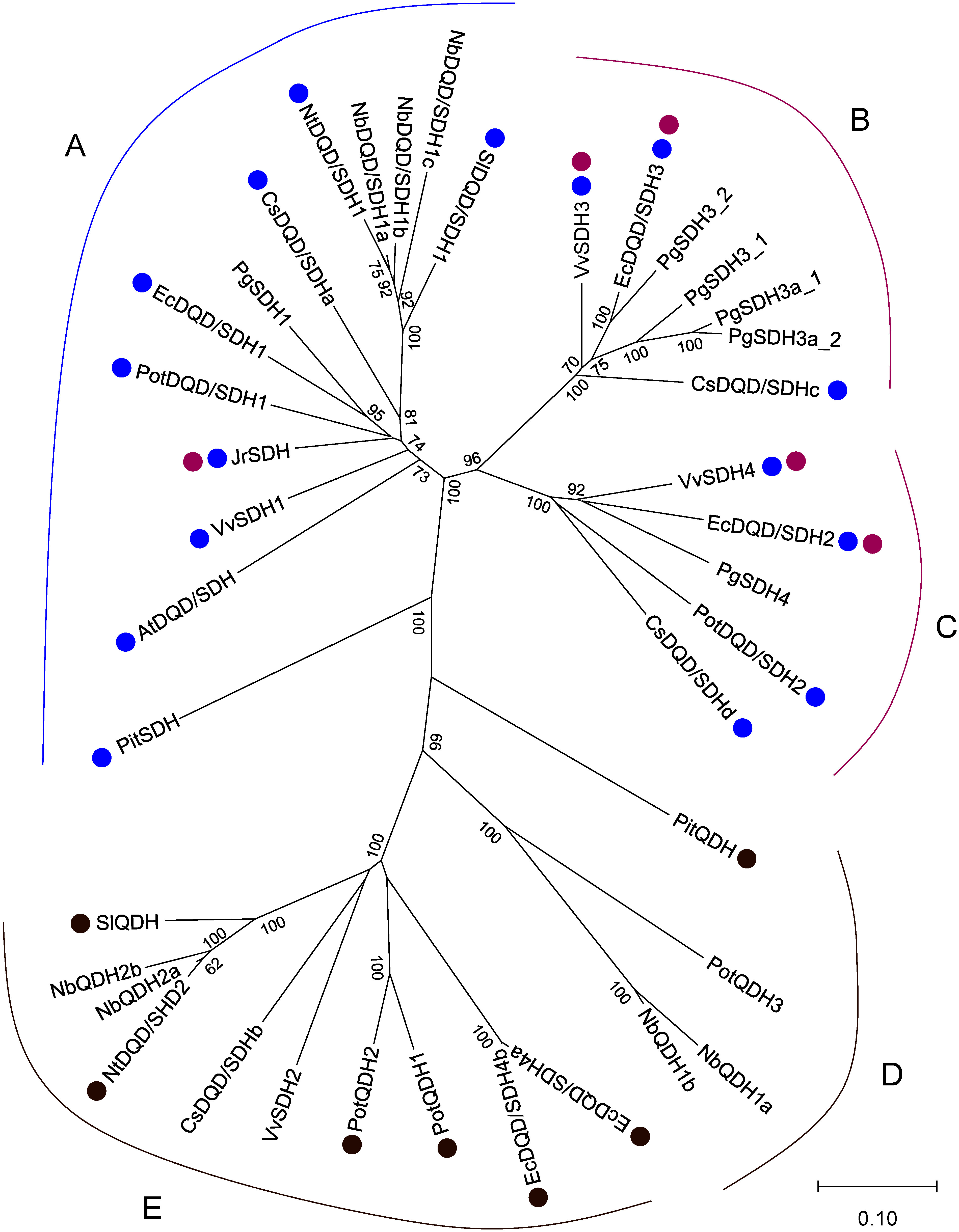
Figure 3. Dehydroquinate dehydratase/shikimate dehydrogenases (DQD/SDHs) identified in seed plants. The phylogenetic analysis of 39 DQD/SDH family members was performed on the basis of an alignment of multiple full-length protein sequences according to the neighbor-joining method using MEGA11 (Tamura et al. 2021). The scale bar represents 0.1 fixed mutations per site. Bootstrap values (1,000 replicates) greater than 60% are indicated. The ID numbers of the members are listed in Supplementary Table S1. Magenta dots indicate enzymes with gallic acid formation activity. Blue and brown dots indicate enzymes that mainly exhibit SDH and quinate dehydrogenase (QDH) activities, respectively. At, *Arabidopsis thaliana* (Singh and Christendat 2006); Cs, *Camellia sinensis* (Huang et al. 2019); Ec, *Eucalyptus camaldulensis* (Tahara et al. 2021); Jr, *Juglans regia* (Muir et al. 2011); Nb, *Nicotiana benthamiana* (Oda-Yamamizo et al. 2023); Nt, *Nicotiana tabacum* (Ding et al. 2007); Pit, *Pinus taeda* (Carrington et al. 2018); Pot, *Populus trichocarpa* (Guo et al. 2014); Pg, *Punica granatum* (Habashi et al. 2019); Sl, *Solanum lycopersicum* (Bischoff et al. 2001; Gritsunov et al. 2018); Vv, *Vitis vinifera* (Bontpart et al. 2016).

According to analyses of the increasingly available genome sequences, enzymes mediating gallic acid formation are clustered in DQD/SDH phylogenetic groups B and C, whereas “classical” DQD/SDH enzymes belong to phylogenetic group A (Figure 3; Bontpart et al. 2016; Tahara et al. 2021). Therefore, the *J. regia* enzyme JrSDH, which is a DQD/SDH phylogenetic group A member, should be re-examined regarding its catalytic activities. DQD/SDH phylogenetic group B and C members are found in core eudicots except for campanulids and lamiids, which overlaps with the distribution of HTs in plants (Figure 1 and Supplementary Figure S1). Gallic acid-forming DQD/SDHs in groups B and C probably evolved through duplication and neofunctionalization of a “classical” DQD/SDH in group A.

## Biosynthesis of gallotannins

### Glycosyltransferases

As an essential intermediate of the HT biosynthetic pathway, β-glucogallin serves as an acyl acceptor and donor (Figure 2). The formation of β-glucogallin is catalyzed by a UDP-glucose:gallic acid glucosyltransferase (gallic acid UGT, EC 2.4.1.136), which has been categorized in the UGT84A clade comprising ester-forming UDP-glycosyltransferases.

In earlier studies, Gross (1982, 1983) showed that *Q. robur* and *Quercus rubra* leaf extracts can produce β-glucogallin and the related 1-*O*-glucose esters of several hydroxybenzoic acids (HBAs; C_6_-C_1_) and hydroxycinnamic acids (HCAs; C_6_-C_3_). Since then, gallic acid UGTs have been identified in several plant taxa (Table 1). The UGT84A clade is in phylogenetic group L within UGT family 1 (Drula et al. 2022; http://www.cazy.org/ (Accessed Dec 20, 2024)). UGT84A subfamily members are found in core eudicots, which correlates with the emergence of HTs and DQD/SDH group B and C members (Figure 1 and Supplementary Figure S2). Similar to the common feature of the UGT superfamily, the enzymes in the UGT84A clade carry the plant secondary product glycosyltransferase box (PSPG box), which is a conserved peptide motif that interacts with UDP-glucose (Hughes and Hughes 1994; Mackenzie et al. 1997).

**Table 1:** Table 1. UDP-glycosyltransferases involved in β-glucogallin synthesis.

Gene/Protein	Other name	Species	Nucleotide ID	Protein ID	Reference
UGT84A6	FaGT2	*Fragaria*×*ananassa*	AY663785	AAU09443	Schulenburg et al. 2016
UGT84A13		*Quercus robur*	KF527849	AHA54051	Mittasch et al. 2014
UGT84A22	CsUGT84A22	*Camellia sinensis*	KP682362	ALO19890	Cui et al. 2016
UGT84A23		*Punica granatum*	KT159805	ANN02875	Ono et al. 2016
UGT84A24		*Punica granatum*	KT159807	ANN02877	Ono et al. 2016
UGT84A25a		*Eucalyptus camaldulensis*	LC189069	BBB21213	Tahara et al. 2018
UGT84A25b		*Eucalyptus camaldulensis*	LC189068	BBB21212	Tahara et al. 2018
UGT84A26a		*Eucalyptus camaldulensis*	LC189071	BBB21215	Tahara et al. 2018
UGT84A26b		*Eucalyptus camaldulensis*	LC189070	BBB21214	Tahara et al. 2018
UGT84A44	VvGT1	*Vitis vinifera*	JN164679	AEW31187	Khater et al. 2012
	VvGT2	*Vitis vinifera*	JN164680	AEW31188	Khater et al. 2012
	VvGT3	*Vitis vinifera*	JN164681	AEW31189	Khater et al. 2012
UGT84A73	JrGGT1	*Juglans regia*			Saxe et al. 2021
UGT84A74	JrGGT2	*Juglans regia*			Saxe et al. 2021

UGT84A25a/b and UGT84A26a/b may represent two pairs of allelic variants.

Enzyme assays with recombinant proteins revealed the substrate promiscuity of gallic acid UGTs for phenolic acids. Thus, UGT84A22 from *C. sinensis* and UGT84A23 and 24 from *P. granatum* do not discriminate between HBA and HCA substrates. According to a previous study, the UGT84A22 activity is highest when gallic acid and *p*-coumaric acid are the substrates (Cui et al. 2016). The catalytic efficiencies of UGT84A23 and 24 are highest for gallic acid and sinapic acid. In addition, UGT84A23 and 24 produce flavone and isoflavone glucosides from apigenin, luteolin, and genistein (Ono et al. 2016). Moreover, UGT84A44 (VvGT1), VvGT2, and VvGT3 from *V. vinifera* and UGT84A6 from *Fragaria*×*ananassa* have a slight preference for HBA over HCA substrates (Khater et al. 2012; Schulenburg et al. 2016). On the basis of expression, UGT84A6 in *F.*×*ananassa* was proposed to catalyze β-glucogallin synthesis in green fruits and 1-*O*-cinnamoylglucose synthesis in ripe fruits (Lunkenbein et al. 2006). In contrast, gallic acid UGTs from *Q. robur* (UGT84A13) and *E. camaldulensis* (UGT84A25 and UGT84A26) have a clear preference for HBA over HCA substrates (Mittasch et al. 2014; Tahara et al. 2018).

The specificities of gallic acid UGTs in planta have rarely been demonstrated. In most cases, their involvement in β-glucogallin synthesis was predicted by correlating transcript levels with HT accumulation patterns (Tahara et al. 2018). Ono et al. (2016) observed that simultaneously knocking down *UGT84A23* and *UGT84A24* via RNAi leads to the decreased accumulation of the major HT punicalagin in *P. granatum* hairy root cultures. As demonstrated for UGT84A23 and 24, gallic acid UGTs are typically localized to the cytoplasm, with an optimal pH of approximately 6.0 in vitro (Ono et al. 2016). This localization raises the question of gallic acid export from plastids because gallic acid-forming DQD/SDH enzymes are predicted to be localized to plastids (Tahara et al. 2021).

### Acyltransferases

In a series of position-specific transacylations, β-glucogallin is converted to 1,2,3,4,6-pentagalloylglucose (Grundhöfer et al. 2001; Figure 2). Because the galloyltransferases (EC 2.3.190) catalyzing these reactions use the 1-*O*-glucose ester β-glucogallin as an acyl donor, they were predicted to be serine-carboxypeptidase-like acyltransferases (SCPL-ATs) (Ciarkowska et al. 2019; Mugford and Milkowski 2012). In planta, SCPL-ATs undergo complex post-translational modifications. Therefore, they cannot be produced as active enzymes in bacteria, which has hindered the functional characterization of SCPL-AT candidates (Stehle et al. 2008, 2009).

Several galloyltransferases have been partially purified from *R. typhina* and *Q. robur* and subjected to biochemical analyses (Niemetz and Gross 2005). Although the amino acid sequences of these distinct enzymes have not been released, there is molecular evidence of their functions in plants accumulating PAs with the galloylated flavan-3-ol structure. In *Diospyros kaki*, Ikegami et al. (2007) identified two putative SCPL-ATs (DkSCPL1 and DkSCPL2) whose expression in fruits is correlated with the galloylated flavan-3-ol content (Akagi et al. 2009, 2011). For *V. vinifera*, genetic mapping and transcriptome analyses identified a QTL related to PA galloylation on chromosome 3, while also producing evidence of a candidate gene encoding a galloyltransferase (VvGAT-like) (Carrier et al. 2013; Huang et al. 2012). Bontpart et al. (2018) combined a genome-wide search for SCPL sequences with a transcriptome analysis and the overexpression of the PA-specific transcription factor genes *MybPA1* and *MybPA2* in hairy roots to identify VvGAT1, VvGAT2, and VvSCP5 as candidate enzymes involved in PA galloylation. These results are consistent with the findings of another study that overexpressed a gene encoding a similar Myb factor, which revealed VvGAT1 and VvGAT2 as important enzymes (Terrier et al. 2009). The genes encoding VvGAT1, VvGAT2, and VvSCP5 are reportedly clustered in the same region of chromosome 3 as the QTL related to PA galloylation (Huang et al. 2012). Liu et al. (2012) purified an epicatechin galloyltransferase from *C. sinensis*, but did not provide the protein sequence. Yao et al. (2022) analyzed this galloyltransferase activity and identified CsSCPL4 and CsSCPL5 as contributing enzymes. Notably, using transgenic *Nicotiana benthamiana* plants, they determined that the galloyltransferase activity is dependent on the co-expression of *CsSCPL4* and *CsSCPL5* and the subsequent interaction between the encoded proteins. In this complex, CsSCPL4 provides the catalytic activity, whereas CsSCPL5, which lacks a functional catalytic triad, is a non-catalytic companion paralog. Similarly, according to co-expression experiments in *N. benthamiana*, DkSCPL1 and DkSCPL2 function as galloyltransferases in persimmon. Furthermore, replacing the N-terminal signal peptides of VvGAT1 and VvGAT2 from *V. vinifera* with the corresponding signal peptides of CsSCPL4 and CsSCPL5 from *C. sinensis* results in chimeric proteins that function as galloyltransferases when co-expressed in *N. benthamiana*.

## Biosynthesis of ellagitannins

The initial ellagitannin in the pathway, tellimagrandin II, is synthesized by the O_2_-dependent oxidation of 1,2,3,4,6-pentagalloylglucose (Figure 2). A laccase-type phenol oxidase (EC 1.10.3.2) purified from *Tellima grandiflora* was observed to catalyze the formation of the 4,6-*O*-HHDP group via the oxidative linkage between the 4- and 6-*O*-galloyl groups (Niemetz et al. 2001; Niemetz and Gross 2003a; Supplementary Figure S1). Niemetz et al. (2001) also detected an enzyme in *T. grandiflora* that catalyzes the oxidation of 2- and 3-*O*-galloyl groups to form a 2,3-*O*-HHDP group, which is a constituent of ellagitannins, including casuarictin and pedunculagin.

For the biosynthesis of ellagitannins, such as tellimagrandin I and pedunculagin, the 1-*O*-galloyl group must be removed from the glucose core (Figure 2), but the enzyme catalyzing this 1-*O*-degalloylation has yet to be identified. Putative candidates are esterases that hydrolyze the carboxylic ester bond between the glucose core and the 1-*O*-galloyl group. Esterases from some plant species, including *Q. robur*, *C. sinensis*, and *F.*×*ananassa*, can catalyze the hydrolysis of galloylglucose esters (Dai et al. 2020; Niehaus and Gross 1997). Another possibility is that acyltransferases transfer the 1-*O*-galloyl group to an acceptor substrate. Denzel and Gross (1991) showed that an acyltransferase extracted from *R. typhina* can use di-, tri-, and tetragalloylglucoses as galloyl donors, implying that acyltransferases can accept a wide range of galloyl donor substrates besides β-glucogallin.

The ellagitannin eucalbanin B identified in *Eucalyptus alba* (Yoshida et al. 1992) has a dimeric structure derived from two tellimagrandin I molecules that are connected by a valoneoyl group (Figure 2) formed by an intermolecular C-O bond between the 4,6-*O*-HHDP group on glucose I and the 2′-*O*-galloyl group on glucose II (Supplementary Figure S1). One of the main products in *E. camaldulensis* is the dimeric ellagitannin oenothein B, which has a macrocyclic structure that links the 4′,6′-*O*-HHDP and 2-*O*-galloyl groups of eucalbanin B to form an additional valoneoyl group (Figure 2 and Supplementary Figure S1). In the first valoneoyl group, the ring of the HHDP group at the O-6 position (ring B in Supplementary Figure S1) is coupled with the galloyl group, whereas in the second valoneoyl group, the ring at the O-4 position (ring B′ in Supplementary Figure S1) is coupled with the galloyl group. An O_2_-dependent laccase-type phenol oxidase purified from *T*. *grandiflora* catalyzes the formation of a 2,4′,6′-*O*-valoneoyl group through the oxidative coupling between the O-4 ring of the 4′,6′-*O*-HHDP group and the 2-*O*-galloyl group of two tellimagrandin II molecules (Niemetz and Gross 2003b; Niemetz et al. 2003). The oxidative coupling between the O-6 ring of the 4,6-*O*-HHDP group and the 2′-*O*-galloyl group may be catalyzed by an unidentified laccase-type phenol oxidase.

## Reconstitution of the HT biosynthetic pathway in model plants

Plants produce a substantial variety of natural compounds. These specialized metabolites with diverse bioactive properties have proven or potential beneficial uses (Birchfield and McIntosh 2020). For example, they have been widely exploited as perfumes, health-promoting supplements, and pharmaceuticals (Atanasov et al. 2015). However, the extraction of these compounds in sufficient quantities is often difficult because of their relatively low abundance in plant species and the technical challenges associated with their purification. Biotechnological engineering using heterologous plant-based production systems may overcome these difficulties.

We recently attempted to reconstitute the HT biosynthetic pathway in *N. benthamiana* via *Agrobacterium tumefaciens*-mediated transient expression (Oda-Yamamizo et al. 2023). *N. benthamiana* lacks the DQD/SDHs in the gallic acid-forming clades (groups B and C in Figure 3), although it has DQD/SDHs from the other groups. The heterologous co-expression of *EcDQD*/*SDH2* and *3* from *E. camaldulensis* in *N. benthamiana* leaves using a polycistronic expression vector (Zhang et al. 2019) leads to the accumulation of gallic acid. Furthermore, if *E. camaldulensis UGT84A25* and *26* are co-expressed with *EcDQD*/*SDH2* and *3*, the transgenic leaves produce β-glucogallin. These results reflect the successful reconstitution of gallic acid production as well as the first committed step (i.e., β-glucogallin formation) in the HT biosynthetic pathway in a plant species that does not naturally accumulate HTs. However, the long-term expression of *EcDQD*/*SDH*s and *UGT84A*s leads to wilting and necrosis at the infiltration site, most likely because of the harmful effects of the accumulation of β-glucogallin beyond a critical concentration. In transgenic *N. benthamiana* leaves, endogenous UGTs catalyze the conversion of gallic acid to 3-glucogallic acid and 4-glucogallic acid. This glucosylation by endogenous UGTs may help to protect *N. benthamiana* leaf cells against the deleterious effects of the reactive intermediate gallic acid.

Genes encoding the enzymes involved in the formation of the third and subsequent intermediates have rarely been identified. Recently, we developed a reliable heterologous expression system for the production of the early intermediates of the HT biosynthetic pathway. This system may be useful for screening and identifying candidate genes encoding the downstream enzymes mediating HT biosynthesis, including galloyltransferases. Hence, it will accelerate the elucidation of the HT biosynthetic pathway as well as the physiological characterization of individual metabolic intermediates produced during the biosynthesis of oenothein B.

## Abbreviations

DQD: dehydroquinate dehydratase
HBA: hydroxybenzoic acid
HCA: hydroxycinnamic acid
HHDP: hexahydroxydiphenoyl
HT: hydrolyzable tannin
PA: proanthocyanidin
SCPL-AT: serine carboxypeptidase-like acyltransferase
SDH: shikimate dehydrogenase
3-DHS: 3-dehydroshikimic acid
UGT: UDP-glycosyltransferase

## Description of Supplementary Files

Supplementary Table S1. Information regarding the DQD/SDHs presented in Figure 3.

Supplementary Figure S1. Phylogenic relationship of DQD/SDHs from 31 different angiosperm orders.

Supplementary Figure S2. Phylogenic relationship of UGTs from 31 different angiosperm orders.
