# Supplementary material for: Elucidation and reconstitution of hydrolyzable tannin biosynthesis
Source: Plant Biotechnol (Tokyo). 2024 Sep 25;41(3):203–12. doi: 10.5511/plantbiotechnology.24.0601a (PMC11921145; doi:10.5511/plantbiotechnology.24.0601a)
Supplement: Supplementary Data [file plantbiotechnology-41-3-24.0601a-s001.pdf]

Supplementary Table S1. Information regarding the DQD/SDHs presented in Figure 3.

Species	Protein name	ID	Reference
Arabidopsis thaliana	DQD/SDH	AT3G06350	Singh and Christendat 2006
Camellia sinensis	DQD/SDHa	AYP64306	Huang et al. 2019
	DQD/SDHb	AYP64307	
	DQD/SDHc	AYP64308	
	DQD/SDHd	AYP64309	
Eucalyptus camaldulensis	DQD/SDH1	BBL52470	Tahara et al. 2021
	DQD/SDH2	BBL52471	
	DQD/SDH3	BBL52472	
	DQD/SDH4a (QDHa)	BBL52473	
	DQD/SDH4b (QDHb)	BBL52474	
Juglans regia	SDH	AAW65140	Muir et al. 2011
Nicotiana benthamiana	DQD/SDH1a	Niben101Scf02709g04001	Oda-Yamamizo et al. 2023
	DQD/SDH1b	Niben101Scf04146g01023	
	DQD/SDH1c	Niben101Scf04216g05005	
	QDH1a	Niben101Scf01642g01015	
	QDH1b	Niben101Scf00247g04007	
	QDH2a	Niben101Scf01740g12012	
	QDH2b	Niben101Scf06613g05001	
Nicotiana tabacum	DQD/SDH1	AAS90325	Ding et al. 2007
	DQD/SDH2	AAS90324	
Pinus taeda	SDH		Carrington et al. 2018
	QDH		
Populus trichocarpa	DQD/SDH1 (Poptr1)	Potri.010G019000.2	Guo et al. 2014
	DQD/SDH2 (Poptr5)	Potri.013G029800.1	
	QDH1 (Poptr2)	Potri.013G029900.2	
	QDH2 (Poptr3)	Potri.005G043400.1	
	QDH3 (Poptr4)	Potri.014G135500.3	
Punica granatum	SDH1	GBGR01003923.1	Habashi et al. 2019
	SDH3_1	OWM62977.1	
	SDH3_2	OWM85406.1	
	SDH3a_1	OWM62975.1	
	SDH3a_2	OWM62976.1	
	SDH4	OWM85405.1	
Solanum lycopersicum	DQD/SDH1	AAC17991	Bischoff et al. 2001
	QDH	Solyc10g038080.1.1	Gritsunov et al. 2018
Vitis vinifera	SDH1	KU163040	Bontpart et al. 2016
	SDH2	KU163041	
	SDH3	KU163042	
	SDH4	KU163043	

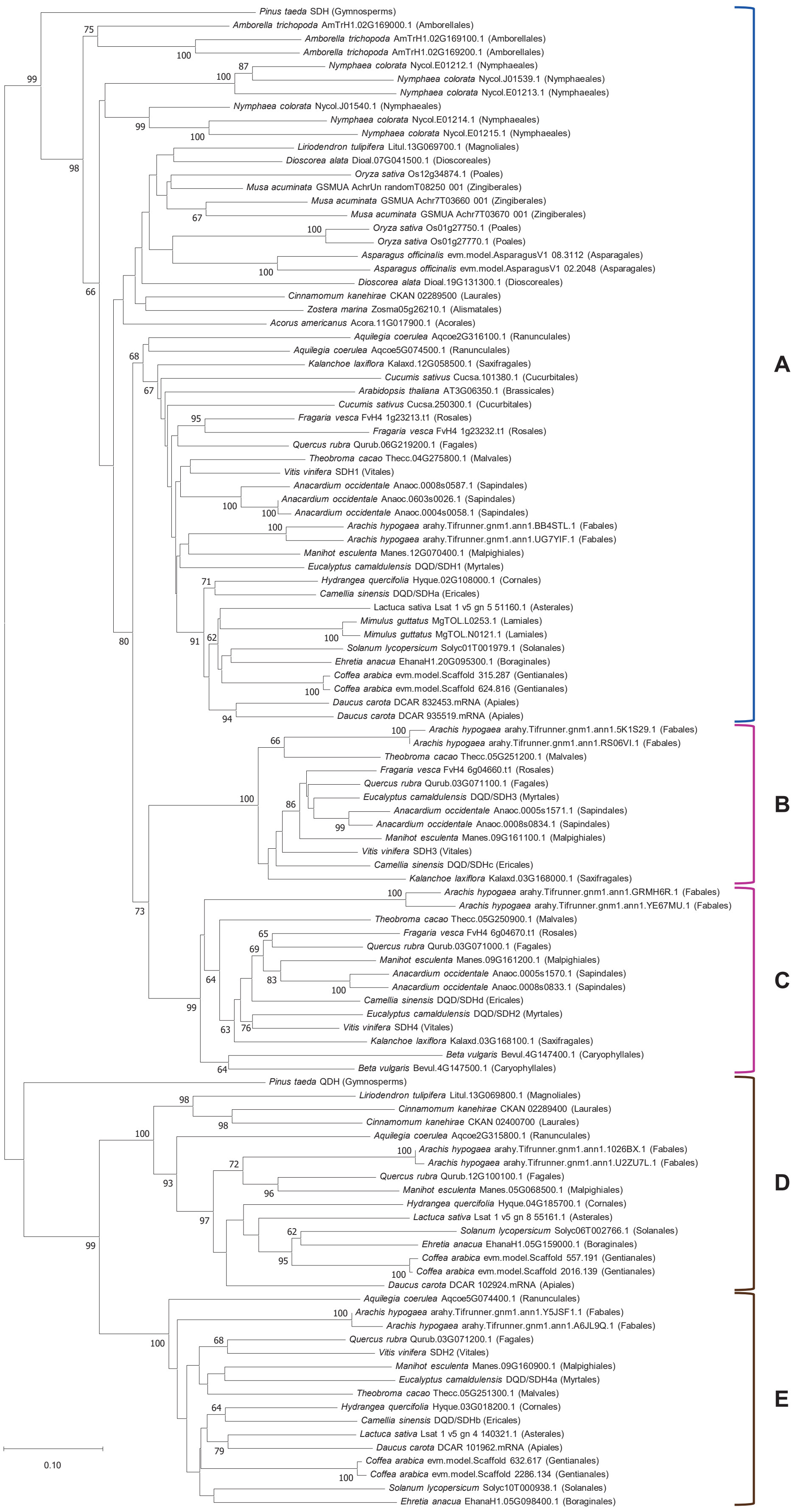

Supplementary Figure S1. Phylogenic relationship of DQD/SDHs from 31 different angiosperm orders. DQD/SDH family members listed in Table S1 or available on the Phytozome database (https://phytozome-next.jgi.doe.gov/) were analyzed using the neighbor-joining method under the same conditions as described in the legend of Figure 3.

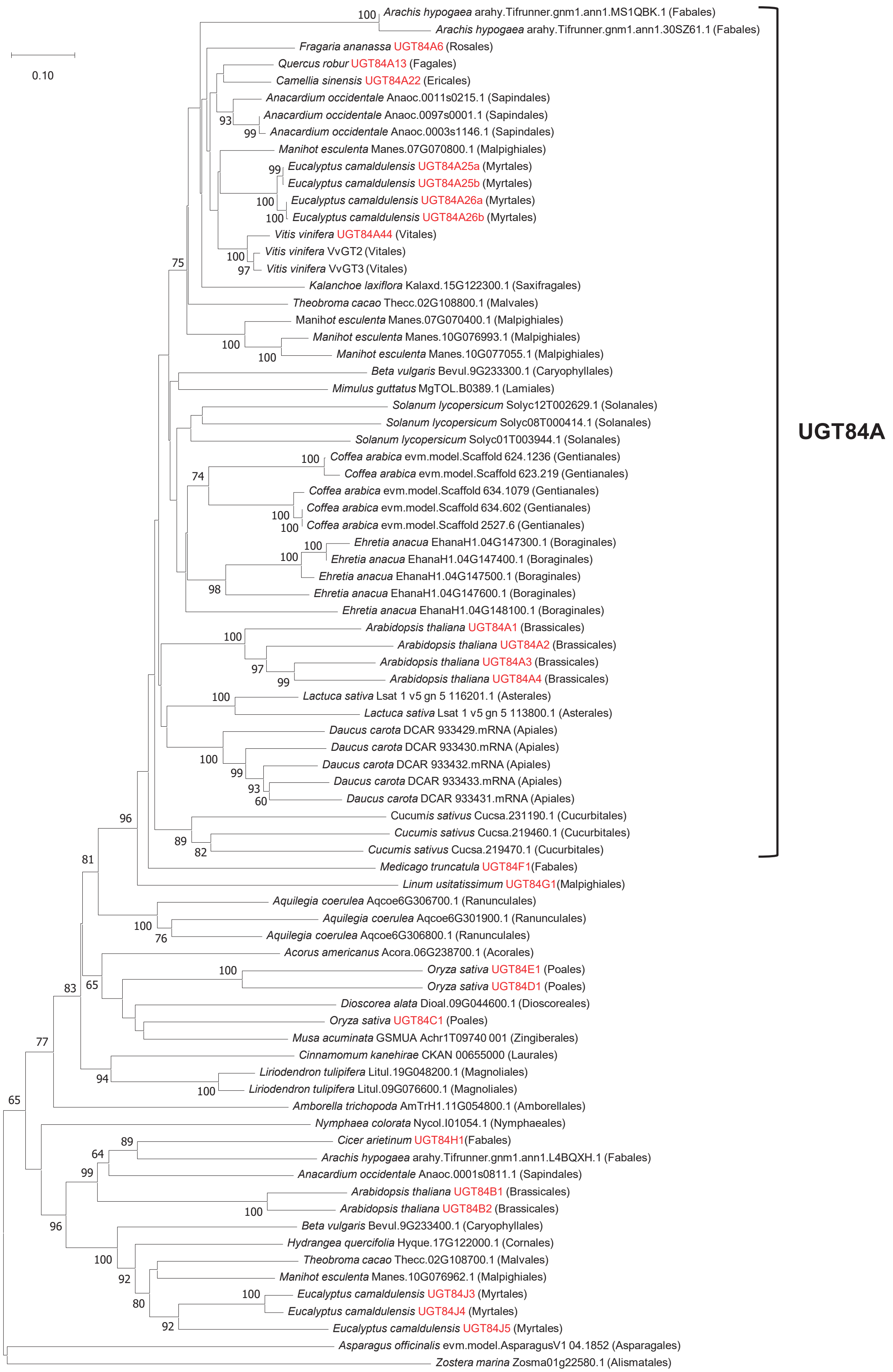

Supplementary Figure S2. Phylogenic relationship of UGTs from 31 different angiosperm orders. UGT family members listed in Table 1, or those available on the Phytozome database (https://phytozome-next.jgi.doe.gov/) or the UGT Nomenclature Committee (https://labs.wsu.edu/ugt/), were analyzed using the neighbor-joining method under the same conditions as described in the legend of Figure 3. Proteins with the highest sequence similarity to UGT84A25a were found from the Phytozome database for each species. UGTs that have been named by the UGT Nomenclature Committee are indicated in red.
